# Risk of non-cancer respiratory diseases attributed to humidifier disinfectant exposure in Koreans: age-period-cohort and differences-in-difference analyses

**DOI:** 10.4178/epih.e2025006

**Published:** 2025-02-22

**Authors:** Jaiyong Kim, Kyoung Sook Jeong, Seungyeon Heo, Younghee Kim, Jungyun Lim, Sol Yu, Suejin Kim, Sun-Kyoung Shin, Hae-Kwan Cheong, Mina Ha, Epidemiology Expert Committee for Reviewing the Epidemiological Correlations between Humidifier Disinfectant Exposure and Health Effects

**Affiliations:** 1 Department of Big Data Research and Development, National Health Insurance Service, Wonju, Korea; 2 Department of Occupational and Environmental Medicine, Wonju Severance Christian Hospital, Yonsei University Wonju College of Medicine, Wonju, Korea; 3 Environmental Health Big Data & Environmental Health Center, Wonju Severance Christian Hospital, Wonju, Korea; 4 Humidifier Disinfectant Health Center, Environmental Health Research Department, National Institute of Environmental Research, Incheon, Korea; 5 Environmental Health Research Department, National Institute of Environmental Research, Incheon, Korea; 6 Department of Social and Preventive Medicine, Sungkyunkwan University School of Medicine, Suwon, Korea; 7 Department of Preventive Medicine, Dankook University College of Medicine, Cheonan, Korea

**Keywords:** Humidifier disinfectants, Respiratory diseases, Population attributable risk fraction

## Abstract

**OBJECTIVES:**

Humidifier disinfectants (HDs) were sold in Korea from 1994 until their recall in 2011. We examined the incidence patterns of 8 respiratory diseases before and after the HD recall and estimated the attributable risk in the Korean population.

**METHODS:**

Using National Health Insurance data from 2002 to 2019, we performed age–cohort–period and differences-in-diffference analyses (comparing periods before vs. after the recall) to estimate the population-attributable fraction and the excess number of episodes. The database comprised 51 million individuals (99% of the Korean population). The incidence of 8 diseases—acute upper respiratory infection (AURI), acute lower respiratory infection (ALRI), asthma, pneumonia, chronic sinusitis (CS), interstitial lung disease (ILD), bronchiectasis, and chronic obstructive pulmonary disease (COPD)—was defined by constructing episodes of care based on patterns of medical care and the clinical characteristics of each disease.

**RESULTS:**

The relative risks (RRs) for AURI, ALRI, asthma, pneumonia, CS, and ILD were elevated among younger individuals (with an RR as high as 82.18 for AURI in males), whereas chronic conditions such as bronchiectasis, COPD, and ILD showed higher RRs in older individuals. During the HD exposure period, the population-attributable risk percentage ranged from 4.6% for bronchiectasis to 25.1% for pneumonia, with the excess number of episodes ranging from 6,218 for ILD to 3,058,861 for CS. Notably, females of reproductive age (19-44 years) experienced 1.1-9.2 times more excess episodes than males.

**CONCLUSIONS:**

This study provides epidemiological evidence that inhalation exposure to HDs affects the entire respiratory tract and identifies vulnerable groups.

## GRAPHICAL ABSTRACT

[Fig f4-epih-47-e2025006]

## Key Message

• From 2002 to 2012, humidifier disinfectant inhalation exposure contributed to 4-25% of respiratory diseases in Korea, with greater effects among younger people, especially for asthma, pneumonia, and interstitial lung disease.

• Older individuals showed more excess episodes for chronic conditions like bronchiectasis and interstitial lung disease.

• The young, the elderly, and reproductive-age women were identified as vulnerable groups in association with humidifier disinfectant exposure.

## INTRODUCTION

In 2006, several hospitals simultaneously reported cases of unexplained respiratory failure in children to academic societies [1]. Subsequently, annual outbreaks of acute interstitial pneumonia among children were observed [2]. In 2009, health authorities conducted microbial testing but failed to identify a microbial cause. Then, in 2011, an outbreak of acute respiratory failure in pregnant female at a tertiary hospital prompted an epidemiological investigation. Therefore, the government identified humidifier disinfectants (HDs) as a likely cause of severe pneumonia with respiratory failure and ordered their market withdrawal in November of that year. HDs are biocidal products designed to prevent scale formation and microbial growth in humidifier water, and they were sold in Korea from 1994 to 2011.

It is estimated that approximately 9.5 million units of HDs, manufactured by various companies, were sold over 17 years, and roughly 2.8 million people (5.69% of the Korean population) used these products. Higher usage rates were observed among children around 5 years old, young adults of reproductive age, and the elderly (65 years or older) [3]. The chemicals in HDs were diverse, primarily including polyhexamethylene guanidine (PHMG, 46.56%), chloromethylisothiazolinone/methylisothizolinone (CMIT/MIT, 26.86%), and others (26.42%) [3]. These chemicals are highly reactive and display bactericidal activity by causing oxidative damage or by physically disrupting the phospholipid structure of cell membranes. Similar toxic effects may also occur in mucosal tissues, including the lungs, eyes, and skin, upon exposure [4].

Ultrasonic humidifiers generate aerosols or gases containing HD chemicals, which humans primarily inhale through the respiratory tract [5]. Most of these aerosols are less than 1 *μ*m in size and are respirable, allowing them to deposit deep within the alveoli [4,6]. Therefore, it is reasonable to expect that exposure to HDs may cause damage throughout the respiratory tract.

The present study aimed to examine the epidemiological patterns of 8 respiratory diseases before and after the recall of HDs and to assess the association between HD exposure and these diseases in the entire Korean population.

## MATERIALS AND METHODS

### Data source and study population

The study population consisted of Koreans registered in the National Health Information Database (NHID) of the National Health Insurance Service in Korea, which covers more than 99% of the 51 million Korean residents. The NHID has been fully digitized since 2002, and we utilized it as a cohort representing the entire Korean population from 2002 to 2019.

The NHID compiles information from 3 components of the National Health Insurance Service—the insured, the insurer, and the service provider. Consequently, it includes data on health service utilization (such as care episodes, drug prescriptions, and treatment materials) as well as personal information (including dates of birth and death, sex, socioeconomic status, and area of residence). Several of these variables were used in the present study [7].

### Period of humidifier disinfectant exposure

Because respiratory illnesses predominantly occur in winter, we defined the annual interval from July 1 to June 30 of the following year, with the year designated as the one containing January. HDs were mainly used during late fall and early spring (November through April), when indoor air humidity was low. For the differences-in-difference (DID) analysis, we defined the domestic HD exposure period as June 1, 2002 to May 31, 2012, and the non-exposure period as June 1, 2012 to May 31, 2019. Although HDs were first introduced in 1994, the first available digitized NHID records began in 2002; thus, 2002 was the earliest feasible historical reference point.

### Respiratory diseases

We examined 8 respiratory diseases: acute upper respiratory infection (AURI; International Classification of Diseases [ICD] codes J00-J06 and J30), acute lower respiratory infection (ALRI; ICD codes J20-J22), asthma (ICD codes J45-J46), pneumonia (ICD codes J12-J18), chronic sinusitis (CS; ICD code J32), bronchiectasis (ICD code J47), interstitial lung disease (ILD; ICD code J84), and chronic obstructive pulmonary disease (COPD; ICD codes J43-J44) (Table 1).

The selection of these respiratory diseases was based on several considerations detailed elsewhere [8]. Briefly, the diseases chosen were those that occurred more frequently among health damage reporters or the general population before the recall than after, as observed in preliminary analyses using the NHID, or illnesses more commonly reported by health damage reporters during medical examinations or monitoring.

Since the NHID is generated for administrative purposes, recorded claims may not always directly correspond to the actual care provided because of variations in payment systems and care behaviors. Therefore, it is necessary to define an “episode of care” within the claim data for research or policy purposes [9]. We defined an episode of care for each respiratory disease based on an estimated window period during which no claims with the corresponding ICD code were recorded in either outpatient or hospital settings [10]. For asthma, we also considered information on prescribed treatment drugs. Detailed methods, including the statistical analyses used to define each disease, have been described elsewhere [4]. Briefly, the window period was 42 days or 49 days for AURI, ALRI, asthma, pneumonia, and CS, and over 27 years for COPD. For intractable chronic conditions such as bronchiectasis and ILD, the first lifetime episode was considered a unique episode of care. Additionally, the first claim of an episode was excluded if it was accompanied by an influenza infection.

We also categorized disease severity into 5 levels by defining episodes of care differently based on diagnostic classification (i.e., primary, secondary, and other diagnoses) and care setting (outpatient or inpatient) (Supplementary Material 1). We report the results for severity levels 2 or 3 as the main findings, since these yielded the highest estimates in analyses for both sexes. Severity level 3 was applied to AURI (Supplemenraty Material 2.1-2.3), ALRI (Supplemenraty Material 3.1-3.3), asthma (Supplemenraty Material 4.1-4.3), pneumonia (Supplemenraty Material 5.1-5.3) and ILD (Supplemenraty Material 6.1-6.3), while severity level 2 was applied to CS (Supplementary Material 7.1-7.3), bronchiectasis (Supplemetary Material 8.1-8.3) and COPD (Supplementary Material 9.1-9.3).

### Statistical analysis

All analyses were performed separately by sex due to the disproportionate distribution of disease frequency and severity among damage reporters—for instance, the initial fatal HD lung injury (HDLI) cases predominantly involved pregnant females [11]— and because HD use was more common among females aged 20-40 [4].

All models included age, period, birth year, income level (by insurance type), and area of residence as covariates to adjust for potential confounders.

The age–period–cohort (APC) analyses evaluated the contributions of age, period, and cohort effects to social variation, disease etiology, age-related changes, and population characteristics. The inherent collinearity in APC analysis—since age plus cohort equals period—was addressed using a generalized linear mixed model. A hierarchical APC model, incorporating both random and fixed effects, was employed to overcome this identification problem [12]. The model specification is as follows:

log(number of events)= log(population)+*μ*+*αi*+*βj*+*γk*+*ϵij*,

where *μ* represents the overall intercept, *i* age, *j* period, *k* birth cohort, and *ϵij* the error term. The model assumes a Poisson distribution for event counts, and an offset term was used to adjust for population size. This approach allowed for the direct calculation of relative risks (RRs) rather than odds ratios, resulting in a more interpretable measure of risk. Fixed-effect variables in the hierarchical APC model included age, area of residence, and income level (by insurance type), while period and cohort (birth year) were treated as random effects [10].

Analyses were performed using NHID data from July 2002 to June 2019. RRs for the age effect were calculated for each age (from 0 years to 85 years or older) using age 14 as the baseline (RR, 1.0), as this corresponds to the age with the lowest annual total healthcare expenditure, thereby increasing the stability of the analyses. For the period effect, RRs for each year were calculated using a baseline defined such that the sum of the logarithmic RR values for the average episode across all ages and cohorts equals zero. Similarly, cohort effects were calculated as RRs by year of birth (1918-2018), with the baseline defined so that the sum of the logarithmic RR values for the mean occurrence for all birth years (from 2002 to 2018) equals 0.

A DID analysis [13] was conducted to assess the extent to which the policy recalling HDs reduced the occurrence of diseases associated with HD exposure and, conversely, to demonstrate that any increase in disease occurrence prior to the recall was attributable to HD exposure. We defined the period from 2003 to 2012 as the HD exposure period and the period from 2013 to 2019 as the non-exposure period. A binary variable representing the period was constructed, and age-specific RRs comparing the exposure period with the non-exposure period were calculated. This involved distinguishing between the age-specific before–after difference (difference 1) and the average RR of the before–after difference across all ages (difference 2), with the final measure being the product of these 2 differences (difference 1×difference 2). The DID analysis was performed separately for each age and birth year. An example of the DID model by age is as follows:

log(number of event)=log(population)+*μ*+*αi*+*β*(*j*×*i*)+*γk*+*ϵij*,

where, *i* age, *j* period, *k* birth cohort; if the year is between 2003 and 2012 then j= 1 (before HDs recall), or if the year is between 2013 and 2019 then j= 0 (after HDs recall).

Thus, the RRs calculated from the DID model represent the ratio of the occurrence rate in 2013-2019 to that in 2002-2012 for each age, adjusted for the average age effect over the entire period (2003-2019) through *αi*.

Based on the RRs estimated by the DID analysis for the entire Korean population, the population attributable risk fraction (PAR%) for HD exposure for respiratory diseases was calculated by age and cohort using Levin’s formula: (RR−1)/RR [14]. Additionally, the number of excess episodes was calculated by multiplying the PAR% by the number of episodes during the exposure period and summing these values across sexes and by age or birth year.

All statistical analyses were conducted using SAS version 9.4 (SAS Institute Inc., Cary, NC, USA). Graphs were generated using Microsoft Excel LTCS 2021 (Microsoft, Redmond, WA, USA) and OriginPro version 2021 (OriginLab Co., Northampton, MA, USA).

### Ethics statement

Exemption for the study was granted by the Institutional Review Board (IRB No. CR321318, CR322328) of Wonju Severance Christian Hospital.

## RESULTS

### Age–period–cohort effects

**Figure 1.**
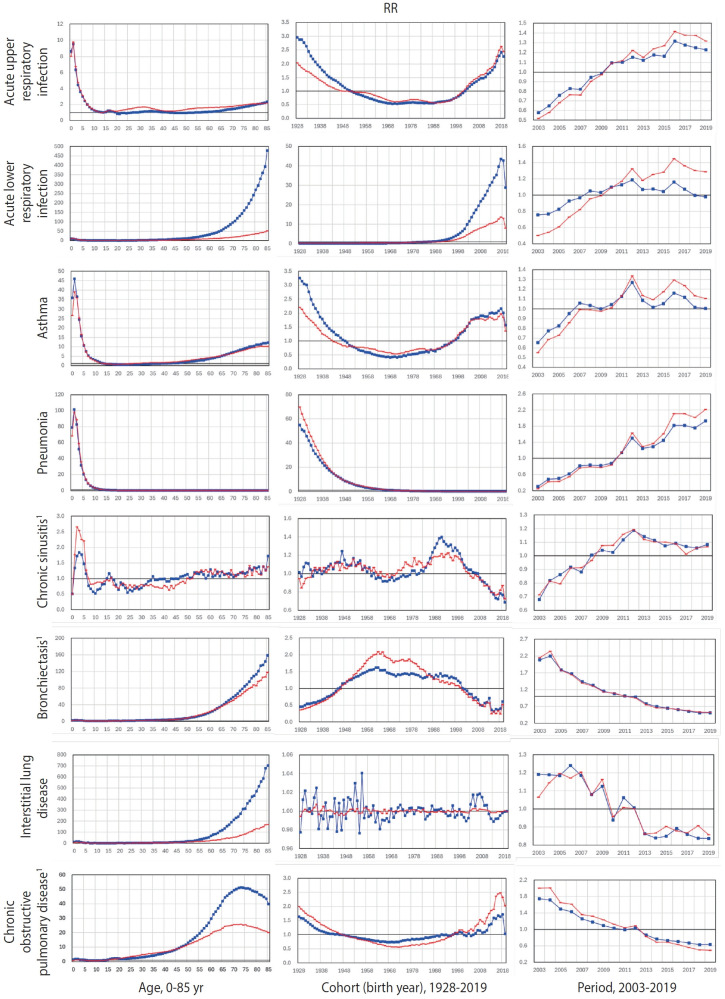
Age-cohort-period effects of 8 diseases of the respiratory system in the total Korean population. International Classification of Diseases codes: acute upper respiratory infection (J00-J06 and J30), acute lower respiratory infection (J32), asthma (J45-J46), pneumonia (J12-J18), chronic sinusitis (J20-J22), bronchiectasis (J47), interstitial lung disease (J84), and chronic obstructive pulmonary diseases (J43-J44). The red line represents females and the blue line represents males. For the age effect, the relative risk (RR) of each episode of care was estimated using age 14 as the baseline; for the period effect, RRs were estimated using a baseline defined such that the sum of the logarithmic RRs across all ages and birth years equals zero; and for the cohort (birth year) effect, RRs were estimated using a baseline where the sum of the logarithmic RR values for the mean occurrence for all birth years from 2002 to 2018 equals zero. All analyses were performed using a generalized linear mixed model. ^1^Severity level (2), otherwise severity level (3).

The results of the APC analyses are presented in Figure 1 and Table 2.

Overall, the 8 respiratory diseases examined generally exhibited higher RRs relative to the reference age effect (i.e., the episode rate at age 14, which was the lowest), ranging from 1.89 to 82.18 in males and from 1.60 to 24.15 in females (Table 2). RRs were notably higher among younger individuals, particularly those aged 0-5 years, for AURI, asthma, pneumonia, and CS, whereas RRs for ALRI, bronchiectasis, ILD, and COPD were elevated among older individuals. Among older age groups, males exhibited higher RRs than females for ALRI, ILD, and COPD (Figure 1). Conversely, among individuals of reproductive age (19-44 years), females had higher RRs than males for AURI, asthma, bronchiectasis, and COPD.

In terms of cohort effects, when compared with the reference (the mean episode rate for all birth cohorts born from 1928 to 2019), cohorts born between 1938 and 2012 exhibited higher overall RRs for ALRI, asthma, pneumonia, CS, and bronchiectasis. These ranged from 1.09 (CS) to 4.39 (ALRI) in males and from 1.00 (asthma) to 3.92 (pneumonia) in females (Table 2). Both the younger generation (born 2000-2012) and the older generation (born 1937-1949) showed high RRs for AURI, asthma, and COPD. In contrast, elevated RRs for ALRI were observed only in the younger generation, while higher RRs for pneumonia were seen only in the older generation (Figure 1).

For period effects from 2003 to 2019, when compared with the average rates across all ages and cohorts, RRs increased over time for AURI, ALRI, asthma, and pneumonia. In contrast, CS exhibited a mixed trend—increasing until 2012 and then decreasing—while bronchiectasis, ILD, and COPD displayed a decreasing trend. This suggests a rise in acute diseases and a decline in chronic diseases over time (Table 2). Nevertheless, across all 8 respiratory diseases, RRs peaked around 2011-2012 and declined by 2013 (Figure 1).

**Figure 2.**
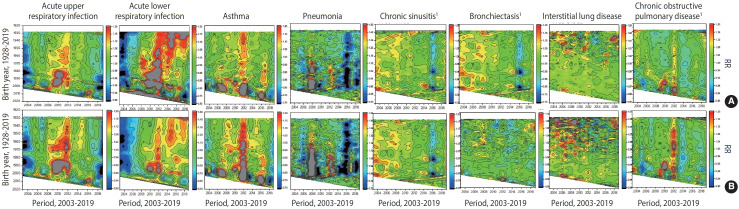
Relative risk (RR) of 8 diseases of the respiratory system as joint effects of cohort (birth year) by period in the total Korean population (A) male, (B) female. International Classification of Diseases codes: acute upper respiratory infection (J00-J06 and J30), acute lower respiratory infection (J32), asthma (J45-J46), pneumonia (J12-J18), chronic sinusitis (J20-J22), bronchiectasis (J47), interstitial lung disease (J84), and chronic obstructive pulmonary diseases (J43-J44). RRs by birth cohort and period were estimated using the interaction term (birth cohort×period) in the corresponding generalized linear mixed model for the age–period–cohort analyses. 1Severity level (2), otherwise severity level (3).

The joint effects of birth cohort and period are shown in Figure 2. Although the distribution of RRs by birth cohort varied among the different respiratory diseases, all birth cohorts exhibited higher RRs around 2012 or earlier, particularly among younger age groups (Figure 2). After 2013, RRs declined for most diseases, with a marked decrease for pneumonia between 2013 and 2016. Additionally, after 2013, higher RRs were observed in older cohorts for ALRI, in females for bronchiectasis, and for ILD, whereas higher RRs were seen in younger cohorts for AURI and asthma around 2016.

### Population-attributable risk % and the number of excess episodes during the humidifier disinfectant exposure period

**Figure 3.**
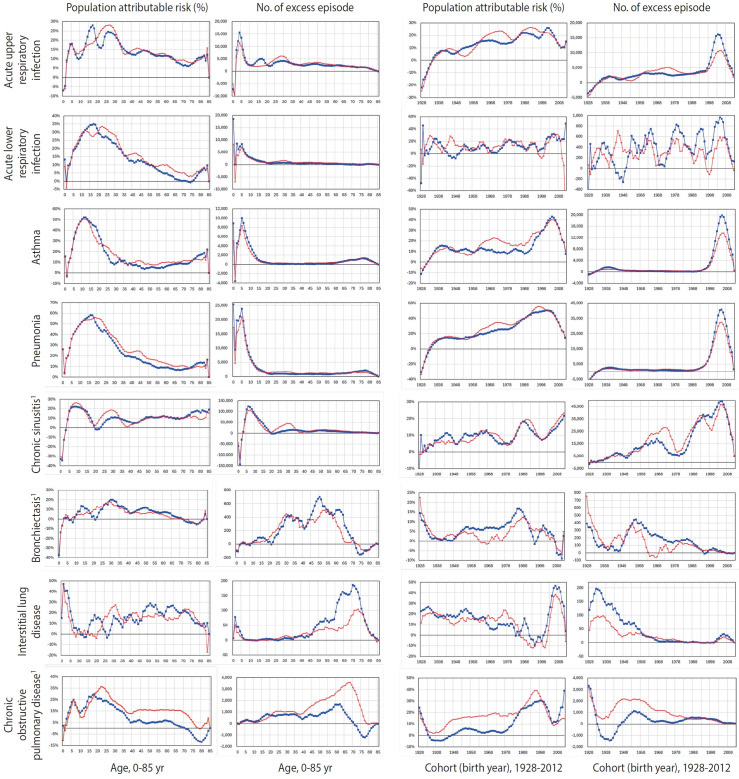
Population-attributable risk fraction (PAR%) and number of excess occurrences of 8 respiratory diseases for humidifier disinfectant exposure in the Korean population. International Classification of Diseases: acute upper respiratory infection (J00-J06 and J30), acute lower respiratory infection (J32), asthma (J45-J46), pneumonia (J12-J18), chronic sinusitis (J20-J22), bronchiectasis (J47), interstitial lung disease (J84), and chronic obstructive pulmonary diseases (J43-J44). The red line represents females and the blue line represents males. Relative risks (RRs) were estimated using differences-in-difference analyses comparing the exposure period (2002-2012) with the non-exposure period (2013-2019), incorporating interaction terms for age×exposure or birth cohort×exposure in the corresponding generalized linear mixed model for the age–period–cohort analysis, using a binary variable for the period. The PAR% was then calculated as (RRt–1)/RRt; number of excess occurrence=*ΣsexΣage((RRt−1)/RRt)×number of case occurred for exposure period*; RRt is RR in the total Korean population. 1Severity level (2), otherwise severity level (3).

The results are shown in Table 3 and Figure 3.

Averaged across all ages, the PAR% during the HD exposure period (2002-2012), compared to the post-recall period (2013-2019), ranged from 5.2% for bronchiectasis to 25.0% for pneumonia. The number of excess care episodes during the 11-year exposure period ranged from 6,267 for ILD to 3,058,861 for CS (Table 3). PAR%s were generally higher among younger age groups (Figure 3), with the highest values observed in the 6-18-year age group, although variations existed: for AURI and bronchiectasis, the peak was in the 19-44-year group, and for ILD, it was in the 0-5-year group (Table 3). Similarly, the highest number of excess episodes by age group mirrored the PAR% pattern: excess episodes for ALRI and pneumonia were highest in the 0-5-year group, while for bronchiectasis and COPD they peaked in the 45-64-year group, and for ILD in the 65-84-year group (Table 3 and Figure 3). Among individuals of reproductive age (19-44 years), females experienced a higher number of excess episodes than males across all 8 respiratory diseases, ranging from 1.1 times (bronchiectasis) to 2.3 times (CS).

Across all cohorts born between 1938 and 2012, during the HD exposure period (2002-2012) compared to the post-recall period (2013-2019), the PAR% ranged from 4.6% for bronchiectasis to 25.1% for pneumonia, and the number of excess episodes ranged from 6,218 for ILD to 2,956,430 for CS (Table 3). Younger generations tended to have higher PAR%s (Figure 3): the highest PAR%s were observed in the youngest cohort (born 2000-2012) for asthma, CS, and ILD; in the second youngest cohort (born 1990-1999) for AURI, pneumonia, and COPD; and in the third youngest cohort (born 1970-1989) for ALRI and bronchiectasis (Table 3). Similarly, the highest number of excess episodes occurred in the youngest cohorts for AURI, ALRI, pneumonia, and CS, and in the second youngest cohort for asthma. However, the highest number of excess episodes was observed for ILD in the oldest cohort (born 1938-1949) and for bronchiectasis and COPD in the second oldest cohort (born 1950-1969). In most disease types, the female-to-male ratio of excess episodes was highest among those born in 1970-1989, ranging from 1.4 (AURI) to 9.2 (asthma), except for bronchiectasis (highest among those born in 1939-1949) and COPD (highest among those born in 1950-1969).

## DISCUSSION

We estimated that HD exposure contributed to 4-25% of the 8 selected respiratory diseases in Korea between 2002 and 2012

For the period from 2002 to 2019 in Korea, younger individuals exhibited a higher risk for acute respiratory conditions—such as AURI, asthma, and pneumonia—whereas older individuals had a higher risk for pneumonia and chronic conditions like ILD and COPD. Additionally, females had a higher risk than males for most respiratory diseases during their reproductive years. Over the period from 2003 to 2019, there was an overall increase in the risk of acute diseases and a decline in the risk of chronic diseases, with a notable peak in RRs around 2011-2012.

The PAR% and the number of excess episodes associated with HD use between 2002 and 2012 varied significantly by age group and disease. Younger individuals generally exhibited higher PAR%s and a greater number of excess episodes for most respiratory diseases, whereas older individuals had higher numbers of excess episodes for chronic conditions such as bronchiectasis, ILD, and COPD. Moreover, females experienced a greater number of excess episodes than males during their reproductive years.

While several analytical epidemiological studies have investigated whether HD use causes disease, to our knowledge, this is the first study to examine the overall impact of HD use on respiratory health across the entire Korean population.

In 2011, a case–control study of acute interstitial pneumonia in perinatal women at a university hospital implicated HDs as a major cause [11]. Since then, case series [15-17] and case-control studies [18,19] have consistently confirmed a strong association between HD exposure and severe lung injury [20]. However, it has been suggested that the health effects of HDs may not be confined to a specific HDLI, but could also manifest as various, including milder, respiratory illnesses [20,21]. Indeed, many HDLI victims experienced a range of respiratory symptoms [21]. This study provides epidemiological evidence linking HD exposure to several non-specific respiratory diseases.

The present study further demonstrates that children, pregnant women, and the elderly are particularly vulnerable to respiratory damage from inhaled HDs. These findings align with existing knowledge. Children have higher metabolic and respiratory rates than adults and consequently inhale more toxicants per unit body weight from contaminated air. Additionally, newborns and infants have immature metabolic systems, and the half-life of chemicals in their bodies is 3-9 times longer than in adults [22]. In pregnant women, increased levels of female hormones—especially progesterone, which acts as a bronchodilator—allow a greater airway surface area to be exposed to respiratory toxicants. Elevated levels of estrogen and prostaglandins E₁ and E₂ further contribute to this bronchodilator effect [23]. Moreover, pregnant women typically have increased lung ventilation compared to non-pregnant adults [24], leading to higher pollutant intake in contaminated environments. In older adults, alterations in the immune cell composition of bronchoalveolar fluid and persistent inflammation in the lower airways result in heightened vulnerability to toxicant exposure and an accelerated decline in lung function [25]. A reduced capacity to combat external toxicants via antioxidant mechanisms [25,26], along with structural changes in the respiratory system and impaired airway mucus clearance [27].

Several aspects of this study strengthen the evidence for a causal relationship between HD exposure and respiratory diseases. First, the study utilized data from the entire Korean population, thereby eliminating the potential for selection bias inherent in sampled data. With over 51 million subjects per year across 18 years (2002-2019), the dataset provided sufficient statistical power that rendered all estimates statistically significant; consequently, confidence intervals were not reported. Second, to control for potential confounders, all analyses were stratified by sex, and models included age, period, birth year, income level, and area of residence as covariates. Third, because influenza infection may serve as a mediator or effect modifier in the association between HD exposure and respiratory disease, episodes of care associated with an influenza infection at onset were excluded from the analyses.

Toxicological studies provide biological plausibility for the causal relationship between HD exposure and respiratory diseases. PHMGs are highly cationic and readily adhere to the negatively charged cell membranes of respiratory epithelial cells, disrupting membrane integrity and impairing cellular homeostasis [28,29]. Once internalized, PHMGs damage lysosomes and mitochondria, leading to cell death [29-34]. PHMGs also induce inflammation [30,32,33, 35,36]. Repeated or excessive exposure can result in the deposition of extracellular matrix proteins such as collagen and fibronectin, leading to airway remodeling [30,36-39]. Animal studies involving PHMG exposure have demonstrated lung inflammatory cell foci, fibrosis, foamy macrophage aggregates, broncho-alveolar hyperplasia, mucus cell hyperplasia, epithelial degeneration/regeneration, congestion, hemorrhage, increased airway resistance, and the secretion of inflammatory and asthma-associated cytokines [40]. Respiratory damage from PHMGs has been observed throughout the respiratory tract—from the upper airways to the lungs— across different species, strains, and exposure methods, with severity increasing in proportion to the exposure dose [40]. CMIT/ MIT reaches respiratory epithelial cells and interacts with intracellular macromolecules by converting into reactive lipophilic species or promoting the generation of reactive oxygen species. It induces cell cycle arrest via p53/p21 activation, thereby limiting cell proliferation [38]. It also increases the expression of proteins involved in mitochondrial membrane depolarization, leading to cell death through oxygen deprivation and inhibition of ATP synthesis [41]. Additionally, it stimulates epithelial cells, resulting in eosinophilic inflammation, and promotes M2 polarization of macrophages along with a sustained increase in transforming growth factor-β, which leads to goblet cell proliferation and mucin overproduction that exacerbates airway inflammation [41]. While systemic or nasal inhalation exposure to CMIT/MIT did not result in lung parenchymal damage in animal studies [42], severe lung parenchymal damage was observed following endotracheal instillation [43]. In the upper respiratory tract, several studies of systemic inhalation exposure have reported inflammation [42,43], and moderate to severe bronchial inflammation was observed in C57BL/6 mice [44]. Although the extent of lung parenchymal damage varied with species, strain, exposure method, and substance concentration, tissue-level findings such as airway remodeling, fibroblastic lesions, and hemorrhage were consistently observed, with severity increasing with exposure dose [40].

Several potential limitations of this study should be considered. First, the NHID does not contain information on individual-level HD use. Nevertheless, the PAR% derived from the DID analysis provides robust evidence for causality based on a counterfactual model comparing pre-recall and post-recall periods within the same population, despite potential confounding from unmeasured factors that may have changed over time. Because the NHID was not originally designed for epidemiological studies, disease diagnoses may be influenced by changes in health insurance policies [4]. Second, acute diseases tend to show an increasing number of episodes over time, whereas chronic diseases such as bronchiectasis, ILD, and COPD show the opposite trend (a period effect). Consequently, the DID analysis comparing recent episodes to those in the past might have underestimated acute diseases and overestimated chronic diseases. However, the consistent pattern of a peak around 2012 followed by a subsequent decline across all diseases strongly indicates the impact of HD exposure.

In summary, during the period from 2002 to 2012, HD exposure contributed to 4-25% of respiratory diseases in the Korean population. The effects were more pronounced among younger individuals or cohorts—particularly for asthma, pneumonia, CS, and ILD—whereas older individuals or cohorts exhibited a higher number of excess episodes for chronic conditions such as bronchiectasis and ILD. Females of reproductive age were more affected than males. These results provide epidemiologic evidence of respiratory health effects throughout the respiratory tract associated with inhalation exposure to HDs and identify the vulnerable populations: the young, the elderly, and females of reproductive age.

## Figures and Tables

**Figure f4-epih-47-e2025006:**
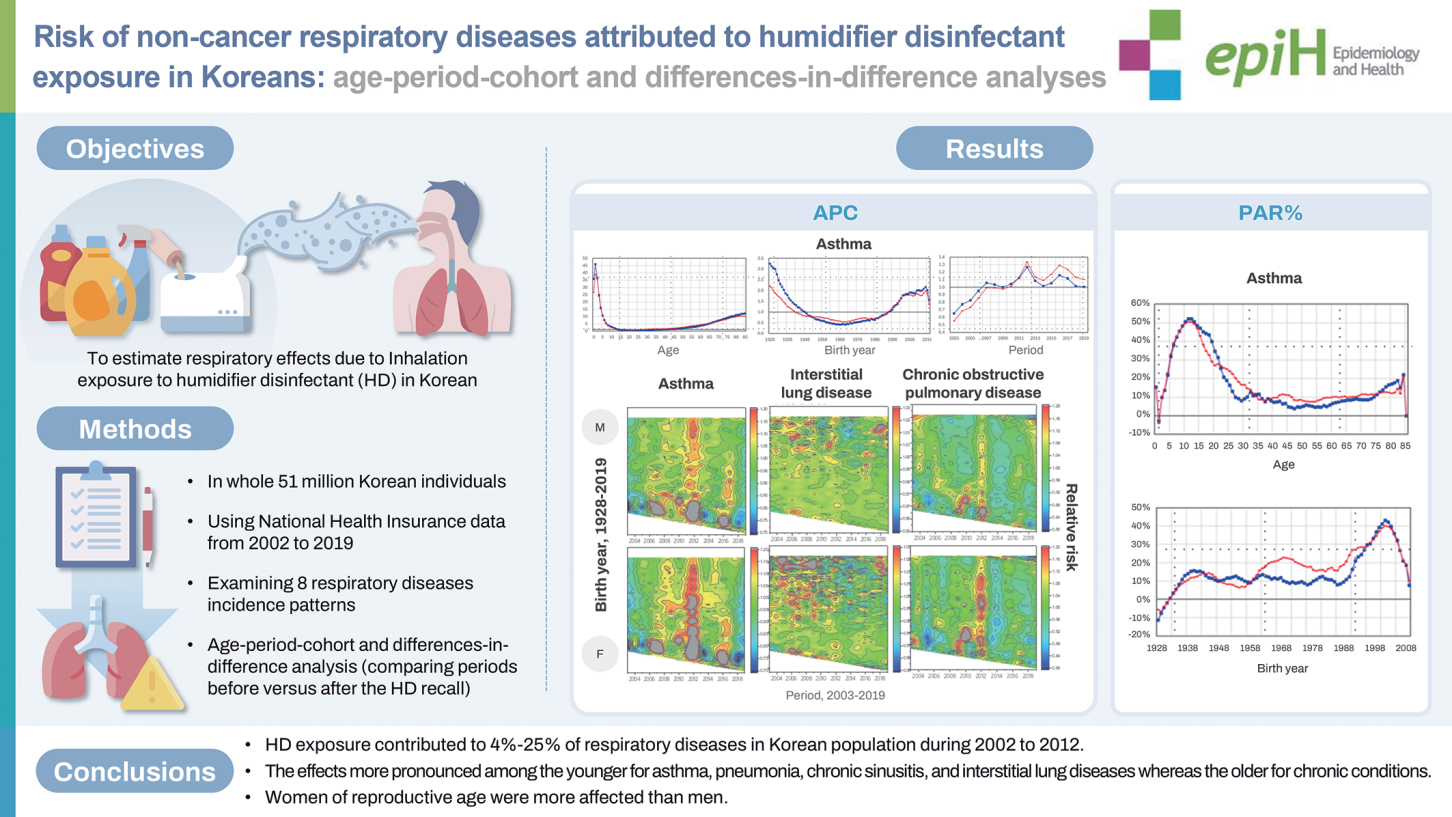


**Table 1.** ICD-10 codes and definition of the episode of care for non-malignant respiratory diseases potentially associated with humidifier disinfectant exposure in the National Health Insurance Data for 2002-2019

Respiratory diseases	ICD-10 codes	Definition of an episode of care [4]
Acute upper respiratory infection	J00-J06, J30	WD 42 days
		+ not accompanied by influenza infection in the initial onset time of an episode of care
Acute lower respiratory infection	J20-J22	WD 49 days
		+ not accompanied by influenza infection in the initial onset time of an episode of care
Asthma	J45-J46	WD 42 days
		+ drugs prescribed for asthma treatment for 1 day or more
Pneumonia	J12-J18	WD 42 days
		+ not accompanied by influenza infection in the initial onset time of an episode of care
Chronic sinusitis	J32	WD 42 days
		+ not accompanied by influenza infection in the initial onset time of an episode of care
Bronchiectasis	J47	The first-appearing J47
		+ not accompanied by influenza infection in the initial onset time of an episode of care
Interstitial lung disease	J84	The first-appearing J84 in observed lifetime
Chronic obstructive pulmonary diseases	J43-J44	WD 9,999 days
		+ not accompanied by influenza infection in the initial onset time of an episode of care

ICD-10, International Classification of Diseases, 10th revision; WD, window periods where no claims are generated with the corresponding ICD-10 codes and can be defined as an episode of care.

**Table 2.** Summary age-cohort-period effects for 8 respiratory diseases in the total Korean population based on the NHID 2002-2019

Diseases	Age effect (vs. episode^1^ rate at the age of 14 yr)			Cohort effect (vs. mean episode^1^ rate of all birth cohorts)			Period effect (vs. mean episode^1^ rates of all ages and birth cohorts)		
	Age (yr)	RR		Birth year	RR		Year	RR	
		Male	Female		Male	Female		Male	Female
Acute upper respiratory infection (J00-J06, J30)	0-5	5.92	6.04	1938-1949	1.48	1.16	2003-2011	0.86	0.82
	6-18	1.28	1.30	1950-1969	0.76	0.87	2012-2019	1.21	1.30
	19-44	1.03	1.40	1970-1989	0.56	0.63	-	-	-
	45-64	1.02	1.48	1990-1999	0.67	0.65	-	-	-
	65-84	1.71	1.93	2000-2012	1.27	1.38	-	-	-
	Total	2.19	2.43	Total	0.95	0.94	-	-	-
Acute lower respiratory infection (J20-J24)	0-5	6.20	7.25	1938-1949	0.19	0.33	2003-2011	0.95	0.82
	6-18	1.24	1.25	1950-1969	0.25	0.44	2012-2019	1.07	1.31
	19-44	3.23	2.25	1970-1989	0.78	0.78	-	-	-
	45-64	22.43	7.69	1990-1999	3.09	1.70	-	-	-
	65-84	193.15	28.38	2000-2012	17.65	6.90	-	-	-
	Total	45.25	9.36	Total	4.39	2.03	-	-	-
Asthma (J45)	0-5	28.24	25.72	1938-1949	1.31	0.99	2003-2011	0.94	0.88
	6-18	2.09	1.94	1950-1969	0.58	0.70	2012-2019	1.09	1.19
	19-44	0.86	1.30	1970-1989	0.55	0.65	-	-	-
	45-64	2.78	3.47	1990-1999	0.95	0.97	-	-	-
	65-84	8.97	8.21	2000-2012	1.75	1.70	-	-	-
	Total	8.59	8.13	Total	1.03	1.00	-	-	-
Pneumonia (J12-J18)	0-5	61.27	62.02	1938-1949	14.57	15.48	2003-2011	0.71	0.66
	6-18	2.64	2.73	1950-1969	3.13	3.41	2012-2019	1.60	1.79
	19-44	0.16	0.20	1970-1989	0.47	0.51	-	-	-
	45-64	0.06	0.05	1990-1999	0.13	0.12	-	-	-
	65-84	0.08	0.03	2000-2012	0.10	0.08	-	-	-
	Total	12.84	13.00	Total	3.68	3.92	-	-	-
Chronic sinusitis (J32)^2^	0-5	4.96	3.75	1938-1949	1.10	1.07	2003-2011	0.89	0.90
	6-18	2.25	1.67	1950-1969	1.02	1.04	2012-2019	1.15	1.14
	19-44	0.66	0.97	1970-1989	1.05	1.05	-	-	-
	45-64	0.82	0.94	1990-1999	1.31	1.19	-	-	-
	65-84	0.77	0.63	2000-2012	0.99	0.97	-	-	-
	Total	1.89	1.60	Total	1.09	1.06	-	-	-
Bronchiectasis (J47)^2^	0-5	2.45	2.42	1938-1949	0.92	0.87	2003-2011	1.53	1.54
	6-18	1.21	1.19	1950-1969	1.46	1.77	2012-2019	0.66	0.66
	19-44	2.05	2.23	1970-1989	1.19	1.35	-	-	-
	45-64	11.38	11.82	1990-1999	1.34	1.16	-	-	-
	65-84	88.63	72.14	2000-2012	0.77	0.70	-	-	-
	Total	21.14	17.96	Total	1.14	1.17	-	-	-
Interstitial lung disease (J84)	0-5	10.29	6.21	1938-1949	1.00	1.00	2003-2011	1.13	1.11
	6-18	1.36	0.88	1950-1969	1.00	1.00	2012-2019	0.87	0.89
	19-44	3.89	3.20	1970-1989	1.00	1.00	-	-	-
	45-64	40.71	17.82	1990-1999	1.00	1.00	-	-	-
	65-84	354.64	92.62	2000-2012	1.00	1.00	-	-	-
	Total	82.18	24.15	Total	1.00	1.00	-	-	-
Chronic obstructive pulmonary disease (J43-J44)^2^	0-5	1.50	1.09	1938-1949	1.03	1.14	2003-2011	1.33	1.48
	6-18	1.20	1.01	1950-1969	0.82	0.75	2012-2019	0.75	0.68
	19-44	2.66	3.84	1970-1989	0.96	0.94	-	-	-
	45-64	15.87	12.36	1990-1999	1.00	0.95	-	-	-
	65-84	47.52	23.94	2000-2012	1.08	1.33	-	-	-
	Total	13.75	8.45	Total	0.98	1.02	-	-	-

Average RR of group of age, birth year, and period was the arithmetic mean of the RR of corresponding each age (years), birth year, or year (period), respectively; For the age effect, the RR of the episode of care of the corresponding disease estimated using age 14 as the referent; for the period effect, those estimated using the referent when the sum of the logarithmic RR for the average occurrence across all ages and birth years equaled 0; for the cohort (birth year) effect, those estimated using the referent when the sum of the logarithmic RR values for the mean occurrence for all birth years from 2002 to 2018 equaled zero; All analyses were performed using a generalized linear mixed model. RR, relative risk; NHID, National Health Insurance Data.

1 Episode of care defined based on a window period of no claim, and other conditions specific for each type of disease.

2 Severity level (2), otherwise severity level (3) among (1)-(5), defined as Stable 1.

**Table 3.** Summary PAR% and estimated number of excess episodes of care for the periods of 2002-2012 compared to 2013-2019 based on differences-in-difference analyses for 8 respiratory diseases in the total Korean population based on the NHID

Diseases	Age (yr)	PAR%			No. of excess episodes			Birth year	PAR (%)			No. of excess episodes		
		Male	Female	All	Male	Female	All		Male	Female	All	Male	Female	All
Acute upper respiratory infection (J00-J06, J30)	0-5	10.5	9.5	10.0	53,746	36,733	90,479	1938-1949	6.5	8.0	7.3	9,310	6,163	15,473
	6-18	17.9	15.6	16.7	51,679	32,439	84,118	1950-1969	13.0	11.7	12.3	52,197	52,437	104,634
	19-44	17.6	20.2	18.9	79,419	100,487	179,906	1970-1989	16.6	21.3	18.9	54,483	78,770	133,253
	45-64	12.5	12.7	12.6	51,510	58,988	110,498	1990-1999	19.6	24.5	22.0	41,635	34,979	76,614
	65-84	8.7	9.6	9.1	29,893	31,894	61,787	2000-2012	17.8	16.2	17.0	144,538	102,062	246,600
	Total	13.8	14.5	14.2	266,248	260,540	526,787	Total	12.2	13.4	12.8	302,163	274,411	576,575
Acute lower respiratory infection (J20-J24)	0-5	11.1	9.5	10.3	49,649	26,141	75,791	1938-1949	4.4	5.4	4.9	-14,682	-10,754	-25,436
	6-18	29.6	27.5	28.6	33,322	27,634	60,956	1950-1969	11.7	11.6	11.7	11,754	14,630	26,384
	19-44	20.2	24.0	22.1	15,774	28,607	44,381	1970-1989	20.3	26.3	23.3	11,961	24,506	36,467
	45-64	7.4	10.1	8.8	8,907	14,813	23,720	1990-1999	18.1	22.8	20.4	13,277	12,910	26,187
	65-84	2.6	5.5	4.1	2,124	7,364	9,488	2000-2012	16.5	13.9	15.2	142,004	101,317	243,321
	Total	13.9	15.9	14.9	109,775	104,560	214,335	Total	10.5	12.6	11.6	164,314	142,610	306,923
Asthma (J45)	0-5	17.5	18.0	17.8	33,923	26,871	60,794	1938-1949	13.4	12.4	12.9	16,952	9,326	26,278
	6-18	47.1	44.1	45.6	40,205	29,600	69,804	1950-1969	11.5	12.9	12.2	3,335	6,125	9,460
	19-44	14.0	16.7	15.4	3,913	7,049	10,962	1970-1989	9.8	18.0	13.9	13,062	120,303	133,365
	45-64	5.5	8.9	7.2	4,776	7,944	12,720	1990-1999	19.7	26.2	23.0	157,027	9,017	166,045
	65-84	12.6	12.1	12.3	18,939	17,020	35,959	2000-2012	32.7	31.7	32.2	11,635	24,992	36,627
	Total	16.7	17.8	17.3	101,756	88,484	190,240	Total	14.4	17.2	15.8	202,011	169,764	371,774
Pneumonia (J12-J18)	0-5	21.2	20.8	21.0	123,113	99,087	222,200	1938-1949	13.8	15.3	14.5	17,062	12,078	29,140
	6-18	52.1	50.6	51.4	79,633	68,425	148,059	1950-1969	17.3	20.1	18.7	22,682	24,479	47,161
	19-44	30.6	36.3	33.4	25,007	37,347	62,354	1970-1989	29.4	34.6	32.0	17,744	28,072	45,816
	45-64	10.5	15.8	13.1	17,797	23,245	41,041	1990-1999	46.8	51.1	48.9	44,550	41,925	86,474
	65-84	10.1	10.0	10.1	30,970	24,022	54,992	2000-2012	39.2	38.7	38.9	362,995	291,786	654,781
	Total	23.7	26.4	25.0	276,520	252,126	528,646	Total	23.8	26.5	25.1	465,034	398,339	863,373
Chronic sinusitis (J32)^1^	0-5	-7.5	-6.5	-7.0	-24,134	-16,963	-41,097	1938-1949	8.7	5.7	7.2	76,365	72,261	148,626
	6-18	18.6	19.3	18.9	936,437	773,223	1,709,660	1950-1969	10.0	8.8	9.4	255,871	303,720	559,591
	19-44	6.1	9.0	7.5	225,160	510,924	736,084	1970-1989	8.8	9.9	9.4	227,796	399,086	626,881
	45-64	10.5	10.4	10.5	202,356	283,909	486,265	1990-1999	11.6	14.2	12.9	358,127	336,328	694,455
	65-84	14.1	10.9	12.5	73,125	94,824	167,949	2000-2012	13.7	15.4	14.5	482,394	444,482	926,876
	Total	10.0	10.3	10.1	1,412,945	1,645,917	3,058,861	Total	9.4	9.5	9.4	1,400,552	1,555,877	2,956,430
Bronchiectasis (J47)^1^	0-5	-5.5	-7.1	-6.3	-56	-33	-90	1938-1949	0.8	2.5	1.7	2,892	5,538	8,430
	6-18	6.3	4.4	5.4	702	355	1,056	1950-1969	6.2	1.8	4.0	6,415	2,994	9,409
	19-44	10.7	11.4	11.1	6,722	7,719	14,440	1970-1989	10.6	5.8	8.2	3,224	1,815	5,039
	45-64	8.6	5.3	7.0	10,546	7,541	18,087	1990-1999	4.3	7.4	5.8	372	476	847
	65-84	-1.0	-1.1	-1.0	51	-500	-449	2000-2012	0.2	-0.2	0.0	220	108	328
	Total	5.7	4.7	5.2	17,964	15,081	33,045	Total	5.3	3.9	4.6	13,123	10,931	24,054
Interstitial lung disease (J84)	0-5	36.6	27.1	31.9	230	134	364	1938-1949	18.1	14.0	16.0	3,003	1,434	4,437
	6-18	4.4	-0.7	1.8	15	1	17	1950-1969	17.1	15.6	16.3	733	550	1,283
	19-44	10.6	15.0	12.8	161	275	436	1970-1989	8.1	12.3	10.2	71	146	217
	45-64	23.5	16.3	19.9	1,509	715	2,225	1990-1999	-2.7	-9.2	-6.0	2	-7	-6
	65-84	15.5	14.8	15.1	2,063	1,163	3,226	2000-2012	30.1	18.3	24.2	222	128	350
	Total	15.7	13.7	14.7	3,979	2,288	6,267	Total	15.5	12.5	14.0	4,031	2,250	6,281
Chronic obstructive pulmonary disease (J43-J44)^1^	0-5	11.6	6.2	8.9	1,019	568	1,587	1938-1949	-2.1	8.5	3.2	-1,400	30,830	29,431
	6-18	21.3	18.4	19.8	4,937	2,769	7,705	1950-1969	4.3	16.3	10.3	12,858	35,354	48,212
	19-44	16.2	23.0	19.6	17,985	25,713	43,699	1970-1989	9.8	19.5	14.7	6,169	14,489	20,659
	45-64	5.6	15.8	10.7	22,604	46,067	68,671	1990-1999	28.0	34.5	31.3	4,881	4,203	9,084
	65-84	-4.3	8.0	1.9	-5,724	32,027	26,303	2000-2012	20.2	15.1	17.6	2,256	1,414	3,671
	Total	9.3	15.9	12.6	40,821	107,144	147,965	Total	10.0	16.9	13.4	24,765	86,292	111,057

The average PAR% and number of excess occurrences for group of age and birth year were the arithmetic mean of the PAF and number of excess occurrences of each corresponding age (years), birth year, or year (period), respectively; RR was estimated through differences-in-difference analyses between exposure (2002-2012) and non-exposure (2013-2019) to humidifier disinfectants, adding the interaction terms of age×exposure or cohort (birth year)×exposure in the generalized linear mixed model for age–period–cohort analysis; PAR%=(RRt−1)/(RRt); number of excess occurrence=Σ*sex*Σ*age*((*RRt*−1)/*RRt*)×*number of case occurred for exposure period*; RRt is RR in total Korean. PAR%, population-attributable risk fraction; NHID, National Health Information Database; RR, relative risk.

1 Severity level (2), otherwise severity level (3) among (1)-(5), defined as Stable 1.

## Supplementary materials

**SM 1.** Severity level classification of the episode of care **SM 2.1.** Age-Cohort-Period effects of acute upper respiratory infection (J00-J06, J30) in total Korean. **SM 2.2.** Relative risk of Acute Upper Respiratory Infection (J00-J06, J30) by cohort and period in total Korean. **SM 2.3.** Risk fraction and number of excess occurrence of acute upper respiratory infection (J00-J06, J30) attributed to humidifier disinfectants exposure in total Korean. **SM 3.1.** Age-Cohort-Period effects of acute lower respiratory infection (J20-J22) in total Korean. **SM 3.2.** Relative risk of Acute Lower Respiratory Infection (J20-J22) by cohort and period in total Korean. **SM 3.3.** Risk fraction and number of excess occurrence of acute lower respiratory infection (J20-J22) attributed to humidifier disinfectants exposure in total Korean. **SM 4.1.** Age-Cohort-Period effects of asthma (J45-J46) in total Korean. **SM 4.2.** Relative risk of Asthma (J45-J46) by cohort and period in total Korean. **SM 4.3.** Risk fraction and number of excess occurrence of asthma (J45-J46) attributed to humidifier disinfectants exposure in total Korean. **SM 5.1.** Age-Cohort-Period effects of pneumonia (J12-J18) in total Korean. **SM 5.2.** Relative risk of Pneumonia (J12-J18) by cohort and period in total Korean. **SM 5.3.** Risk fraction and number of excess occurrence of pneumonia (J12-J18) attributed to humidifier disinfectants exposure in total Korean. **SM 6.1.** Age-Cohort-Period effects of interstitial lung disease (J84) in total Korean. **SM 6.2.** Relative risk of Interstitial Lung Disease (J84) by cohort and period in total Korean. **SM 6.3.** Risk fraction and number of excess occurrence of interstitial lung disease (J84) attributed to humidifier disinfectants exposure in total Korean. **SM 7.1.** Age-Cohort-Period effects of chronic sinusitis (J32) in total Korean. **SM 7.2.** Relative risk of Chronic Sinusitis (J32) by cohort and period in total Korean. **SM 7.3.** Risk fraction and number of excess occurrence of chronic sinusitis (J32) attributed to humidifier disinfectants exposure in total Korean. **SM 8.1.** Age-Cohort-Period effects of bronchiectasis (J47) in total Korean. **SM 8.2.** Relative risk of Bronchiectasis (J47) by cohort and period in total Korean. **SM 8.3.** Risk fraction and number of excess occurrence of bronchiectasis (J47) attributed to humidifier disinfectants exposure in total Korean. **SM 9.1.** Age-Cohort-Period effects of chronic obstructive pulmonary diseases (J43-J44) in total Korean. **SM 9.2.** Relative risk of Chronic Obstructive Pulmonary Disesase (J43-J44) by cohort and period in total Korean. **SM 9.3.** Risk fraction and number of excess occurrence of chronic obstructive pulmonary diseases (J43-J44) attributed to humidifier disinfectants exposure in total Korean.
